# Clinical Notes from the N. Y. Hospital

**Published:** 1892-03

**Authors:** Eugene Clark

**Affiliations:** Lockhart, Texas


### Correspondence.

### CLINICAL NOTES FROM NEW YORK HOSPITALS.

LETTER FROM EUGENE CLARK, M. D., LOCKHART, TEXAS.

*Editor Daniel's Texas Medical Journal:*

I stopped a few days in New Orleans, and visited the Charity Hospital, under the management of Dr. Miles. It keeps pace with the foremost hospitals of America; in its thousand beds are to be found most of the ills that man is heir to. They have just completed two large buildings for the out patient department. I also visited the Eye, Ear, Nose and Throat Hospital, which is a charitable institution, under the management of Dr. A. W. De Roaldes. It is about the only institution of the kind in the South, and compares favorably with those in the East. It is fixed up with all the modern appliances.

I will mention a case in which Dr. Roaldes introduced O'Dwyer's intubating tube. It was a case of almost complete stenosis and synechia of the vocal cords, the result of catarrhal ulceration. It began eleven years ago, with hoarseness, from getting wet, and progressed rapidly until there was complete loss of voice, and the operation was seriously embarrassed. The vocal cords were found to be agglutinated, leaving a very small aperture at the posterior commissure. There was no evidence of tubercular or of syphilitic trouble. During a visit, the patient was taken with an attack of asphyxia, and tracheotomy was performed, the larynx having become air tight. Repeated attempts to pass a small laryngeal probe failed.

After several months, in which repeated trials were made, Dr. De Roaldes succeeded in passing a very small probe from the canula wound in the neck, which emerged in the throat at the seat of the old laryngeal aperture. This was first dilated with threads, until the opening was sufficient to admit a small hard rubber tube. This process was continued, until the O'Dwyer intubating tube could be used. It is hoped to sufficiently dilate the laryngeal stenosis to allow of the withdrawal of the canula, and the permanent closure of the tracheotomy wound.

At the New York Polyclinic, I found Texas well represented. In fact, excepting New York, there were more physicians from Texas than from any other State in the Union.

Prof. Seibert, believing that diphtheria is a local disease dependent upon the presence of the Loeffler bacillus, and finding that he had little success in the treatment of this disease with the ordinary local applications, concluded it was due to the fact that the bacilli are protected by the pseudo-membrane. He devised an instrument with which to introduce the remedies through this membrane, to reach the bacilli in this submucous tissue. It consists of a barrel, like that of the ordinary hypodermic syringe, to which is attached a needle about three inches long, which is flattened out at the end to about the size of a coffee grain, and at right angles from the flat surface five little needles project about a quarter of an inch. With this little instrument, he injects a freshly prepared solution of chlorine water (1-500) into the diseased tissues, and repeats it in from one to three hours, if the temperature be not reduced, and other symptoms of improvement appear.

Dr. Seibert reports that up to this time he has treated 114 cases of diphtheria in this way, and all recovered but two, who had laryngeal diphtheria, and he could not get at the diseased parts.

I saw two operations for ectopic pregnancy; one by Prof. Weir, of the New York Hospital. The woman was brought in in an almost moribund condition. She was anæsthetized, and the abdomen opened; it was found full of blood, the tube having ruptured,—and a three months fœtus was found in the abdominal cavity. The tube and contents were ligated and removed, and the abdomen cleansed.

The other case was in Prof. Tuttle's clinic, at the Roosevelt Hospital. Seven days before the operation, patient was taken with pains, and lost a little blood; afterwards passed a complete membraneous cast of the endometrium. On examination, the uterus was found somewhat enlarged, but empty, and a hard, baggy mass found in the right broad ligament. Ectopic pregnancy was diagnosed. The abdomen was opened, when the tube was found to have been slightly ruptured; there was some peritonitis, and a little blood in the abdomen. The tube was ligated and removed, and the stump touched with a cautery. The fœtus was the size of a small finger. Both cases progressed favorably.

I saw a great many surgical operations, and will give you a short account of those by Prof. McBurney, at the Roosevelt Hospital. The first case was that of a woman with varicose ulcers on both legs. They were about five inches in diameter, and of eight years standing. Dr. McBurney did Thiersch's skin grafting operation. The limb was stripped of blood, and an Esmarch's bandage applied. An incision in the skin about an eighth of an inch from the edge was carried all around the ulcer; —with a sharp knife the granulations of the ulcer were shaved off to the incision in the skin, leaving a clean, healthy surface. Long strips of the superficial layer of the skin were shaved off with a razor, so thin that they were transparent. With these strips the denuded surface was completely covered. The parts were then covered with protective [?] and dressed with wet bichloride gauze. Some days later the ulcers were again dressed, and found to be entirely covered with healthy skin.

He also operated on a young man with a tumor over the left eye, which caused the eye to protrude. A curved incision was made over the left eye, exposing the frontal bone, through which the doctor chiseled, and found an osseous tumor in the frontal sinus about the size of a large walnut. This had caused absorption of the supra orbital plate, and was encroaching upon the eye, and causing it to bulge forward. It had also caused absorption of the posterior wall of the frontal bone, and was adherent to the dura-mater. The tumor was removed, and found to be hard and smooth on the outside, and soft and cancellous on the inside. The wound was packed with gauze, as the hemorrhage was considerable, and not easily controlled. By the next Saturday, the patient was able to walk into the amphitheater to have his wound dressed. It was found to be doing nicely, the eye having returned to its proper position.

The next case was that of a boy fourteen years of age, with the following history: When four years of age he fell, causing a compound fracture of the parietal bone on the left side, near the sagital suture; following this there was an abscess of the brain substance. This was lanced, and was followed by hemiplegia on the right side. There was gradual improvement, but the hemiplagia never entirely disappeared. About fifteen months ago the patient fell on his head again, after which he began to have epileptic seizures,—at first, one every four or five days, but at the time of the operation he had been in the hospital five days, and had had four attacks. They would begin in the hand of the right side, and go up the arm and down the leg on the same side. The surgeon located the lesion at the fissure of Rolando, on the left side. The skin was incised, and with the Rongeur forceps an elliptical piece of skull about two and a half inches long was removed. The dura-mater was then cut through, and a cyst the size of a robins egg was found at the fissure of

Rolando. The cyst contained twenty-five minims of clear fluid. The dura-mater was sewed up, and the wound packed with gauze. The next Saturday the patient was brought in and the wound dressed, and found to be doing nicely. At the last account, there had been no return of the epilepsy.

The next day an operation was made for a subclavian aneurism in third portion. The patient was about thirty years of age. About five years ago he began to notice that the veins in the right arm were full and painful. This gradually increased, until it became so great that he was unable to let the arm hang at his side, and the only rest that he could get was in the recumbent position, with the arm elevated.

A Z-shaped incision was made first along the sterno-cleido mastoid, then along the clavicle, and the other end of the Z going down over the pectoral muscles. The skin was dissected well back out of the way, the clavicle divided over the tumor and pulled up. Some trouble was experienced in exposing the aneurism, as the brachial plexus was adherent to it, and was separated with difficulty. Both ends were ligated, and tumor removed. Patient died.

